# Generalized Anxiety Disorder and Depressive Symptoms among Pakistani Population during the Second Wave of the COVID-19 Pandemic: A Regression Analysis

**DOI:** 10.4269/ajtmh.21-0380

**Published:** 2021-08-30

**Authors:** Farah Yasmin, Kartik Dapke, Muhammad Rahman Khalid, Hina Naz, Farah Naz, Bushra Admani, Muhammad Sohaib Asghar, Sana Awan, Rachana Phadke, Muhammad Saleem

**Affiliations:** ^1^ Department of Internal Medicine, Dow University of Health Sciences, Karachi, Pakistan;; ^2^ Indira Gandhi Government Medical College, Nagpur, India;; ^3^ Department of Adult Cardiology, National Institute of Cardiovascular Diseases (NICVD), Karachi, Pakistan;; ^4^ Department of Internal Medicine, Dr. Ruth K. M. Pfau Civil Hospital Karachi, Karachi, Pakistan;; ^5^ Department of Psychology, Dow University Hospital, Dow University of Health Sciences, Karachi, Pakistan

## Abstract

Over a span of 1 year, with millions infected, COVID-19 has spread to every part of the world and now poses a health threat to each and every one of us. The outbreak has consequently resulted in multiple health problems such as stress, anxiety, depressive symptoms, insomnia, panic, and denial globally. Several factors have contributed to this rising number of psychiatric consults all over the world. The primary objective of this study was to investigate the impact of COVID-19 pandemic on the mental health of Pakistani population during the second wave of the pandemic in this region. We conducted an online web-based cross-sectional survey comprising 500 participants. The questionnaire assessed the demographic information, attitude, and knowledge concerning COVID-19 outbreak in addition to generalized anxiety disorder (GAD) utilizing the GAD-7 scale and depressive symptoms using the Center for Epidemiology Scale for Depression (CES-D) scale. The response rate of the study was 90.9%. The results of the survey indicated a prevalence of 25.4% of GAD, and 18.8% of depressive symptoms. Furthermore, nearly 34.8% of participants feared contracting COVID-19, 62.8% obtained constant critical updates regarding COVID-19, while 17.6% did not understand the knowledge regarding COVID-19. In the multivariate regression models, GAD was significantly associated with gender, age, and checking constantly of critical updates regarding COVID-19. Similarly, participants under 30 years had a higher risk of developing depressive symptoms than those above (> 30 years). Lastly, participants with no formal education were also found to be more prone to developing depression. We identified a potential threat to mental health during the pandemic.

## INTRODUCTION

COVID-19 is an infectious disease caused by a newly discovered strain of coronavirus known as severe acute respiratory syndrome coronavirus-2 (SARS-CoV-2). The first case of SARS-CoV-2 was seen in Wuhan, China, in December 2019 and since then, as a result of its highly infectious nature and rapid human-to-human transmission via respiratory droplets, the number of COVID-19 cases have spread globally at an alarming rate, causing it to be declared as a pandemic by the WHO on March 11, 2020.^1^ Current data states that it has caused more than 771,000 deaths globally, while nearly 21 million cases have been confirmed in at least 215 countries and territories. In addition, nearly 13 million people have recovered to date.^2^

The first COVID-19 case in Pakistan emerged in February 2020, and since then the country has witnessed three waves of the novel disease, which have severely challenged the daily lives of the people as well as the healthcare system of the country.^3^ Initially, the spread of the virus was difficult to contain as there was a mixed response from the general population, owing to the noncompliance of personal protective measures. The COVID-19 vaccination started on February 3, 2021 in Pakistan and currently, 1.8% of the country’s population is fully vaccinated.^4^ According to the statistics provided by National Command and Operation Center (NCOC), the number of deaths from coronavirus has risen to 22,597 across the country, whereas the number of people affected has reached 975,092. As of July 12, the total active COVID-19 cases in Pakistan are 38,622 while the country’s positivity rate stands at 3.84%.^2^ It is alarming to note the rising number of highly contagious delta variant cases in the populous city like Karachi, which already has COVID-19 positivity ratio of 14.58%.^3^

In response to the rising COVID cases, the government responded by introducing a lockdown across the country in early March 2020. To curb the spread of the disease, all channels of communication such as television, radio, newspapers, social media, and so on, were used to spread awareness about the disease, which proved quite beneficial as shown by good public knowledge of COVID-19.^5^ However, the unpredictability of this new disease, as well as preventive measures like self-isolation, social distancing, and quarantine precipitated psychological problems, and predisposed individuals to mental health challenges. An online survey conducted in early stages of the pandemic revealed that approximately 68% of the population was worried about contracting the disease.^6^ Findings of another study suggested that around 78% of the population had anxiety, while half of them were depressed.^7^ Hayat and others determined the impact of the COVID-19 outbreak on mental health status, and demonstrated 24.4% of the participants to suffer from mild to moderate depression, and 30.7% of them to suffer from mild to moderate anxiety.^8^

The demographic characteristics of Pakistan are of low middle-income countries where healthcare services are already scarce. During this time of global health crisis, it is inevitable that mental health challenges will be faced by frontline healthcare workers (HCWs) who are particularly vulnerable in this situation. Inadequate healthcare capacity, fear of contracting and transmitting the virus to friends and family, overburden of work, and stress are some of the key factors that could be harmful to the mental well-being of HCWs. This is supported by multiple studies done in Pakistan, which explored the effect of the pandemic on depression and anxiety among HCWs. A higher prevalence of depression and anxiety was seen in younger physicians, those working in the emergency, and those working in direct contact with COVID-19 cases.^9^ Additionally, studies also showed that greater exhaustion, greater family strain, and reduced feelings of protection had an impact on the levels of anxiety among physicians.^10^ A study conducted by Hayat et al., reported 82.2% of HCWs to use online psychological resources to deal with their psychological distress. Furthermore, they demonstrated female HCWs, nurses, frontline HCWs, and those aged 30–49 years to be at a greater risk of anxiety and depression (*P* < 0.001).^11^ It is also important to note that people with preexisting mental health issues such as anxiety and depression, those living alone such as the elderly, as well as patients suffering from chronic diseases are most vulnerable to developing mental health problems because of COVID-19.^12^ The existing chronic conditions of such patients puts them at a greater risk of developing severe complications from COVID-19, which can increase stress and anxiety, and ultimately exacerbate problems with health and mental well-being.^12^

During these times of crisis, inevitably people are expected to develop fear of the unknown and resist change, the magnitude of which has brought about population-wide psychological impact. This outbreak has resulted in additional psychological health problems such as stress, anxiety, depressive symptoms, insomnia, denial, anger, and fear globally. The unpredictability and uncertainty of the disease, along with information overload, misinformation, and social isolation are some of the important contributing factors to cause mental health problems. The impact of the pandemic on the general population’s mental health has been a subject of multiple recent researches. One such study from China reported that one-third of its participants showed anxiety disorders, nearly one-fifth had depressive symptoms and sleep problems, indicating that the uncertainty of the epidemic progression caused greater psychological pressure on the public.^13^ Another study from the same region showed almost 35% of the respondents to experience psychological distress.^14^ A cross-sectional survey, conducted in India, observed that almost one-fourth (25.1%) of the participants were depressed, 28% were anxious, and 11.6% were stressed.^15^ Evidently, people’s mental health has been negatively affected as well in previous pandemics such as the SARS.^16^ Therefore, the primary aim of this study was to evaluate the prevalence of generalized anxiety disorder (GAD) and depressive symptoms among the Pakistani population during the second wave of the COVID-19 outbreak. A secondary aim was to explore the potential influence factors on the mental health of citizens.

## MATERIALS AND METHODS

An online web-based cross-sectional survey was distributed between January 1, 2021 and January 25, 2021 through social media platforms such as Facebook, WhatsApp, and e-mails. All individuals aged > 15 years of either gender, that is, male or female residing in the four provinces of Pakistan, who had access to Internet and understood Urdu were included in the survey. All individuals aged < 15 years, and who refused to provide informed consent were excluded. The questionnaire was distributed via convenience sampling, and snowball sampling techniques. The sample size of at least 385 individuals was calculated by using the Rao soft digital sample size calculator, with a margin of error of 5%, a 95% CI, a response distribution of 50% to provide maximum variance, and a population size of 225 million. This web-based questionnaire was completely voluntary and noncommercial (https://forms.gle/WmFDDf7uTjcXC4Hi6). To encourage the recruitment of potential participants, all participants in the survey can receive a report on their mental health after completing the evaluation. The questionnaire was delivered in the native language of the population, that is, Urdu language, and was reviewed by two senior physicians for validity and reliability. The questionnaire was further modified based on their recommendations and suggestions. A pilot survey was also conducted on 11 participants that yielded the questionnaire to be comprehensive, easily accessible, understandable, and took approximately 5 minutes to complete.

Citizens who volunteered to participate in the survey were allowed to answer the questionnaire only once, and were given the offer to terminate the survey at free will and at any time they feel uncomfortable within the study period. The participants were also assured that their anonymity and confidentiality would be maintained as all data provided by the participants would be kept confidential. The questionnaire composed of an introductory paragraph briefly stating the aim of the study, description on voluntary participation, declaration of anonymity and confidentiality, and a mandatory informed consent obtained from all participants. Six items of the questionnaire recorded the sociodemographic information, three items accessed the attitude of the participants regarding the COVID-19 outbreak, and seven items recorded the COVID-19 related information taken from a previously conducted study in China.^17^ The participants also completed two standardized questionnaires that accessed GAD and depressive symptoms. The items evaluating the attitude concerning COVID-19 outbreak included the average time spent focusing on the COVID-19 outbreak information every day, obtaining constant critical updates regarding COVID-19, and fear of contracting COVID-19. Knowledge of the COVID-19 was assessed based on the following seven judgment questions about COVID-19-related knowledge: 1) Inhalation of droplets from sneezing, coughing, or talking to an infected person could cause infection; 2) Contact with something contaminated by an infected person could lead to infection; 3) The incubation period of the virus does not exceed 14 days; 4) Contact with an asymptomatic person might also lead to infection; 5) There are already targeted drugs that could cure the disease; 6) Not all people with COVID-19 will develop to severe cases. Only those who are elderly, have chronic illnesses, and obese are more likely to be severe cases; 7) The main clinical symptoms of COVID-19 are fever, fatigue, dry cough, and muscle ache. Each correct answer was awarded a score of one point while no point was given for an incorrect answer. Participants with scores ≥ 5 points, equal to 4 points, and ≤ 3 points were considered to quite understand, generally understand, and do not understand the knowledge of COVID-19.

The Pakistani version of GAD-7 scale was used to access the individual’s anxiety symptoms. The GAD-7 has been previously used in Pakistani populations, and found to be highly reliable (Cronbach’s alpha = 0.92).^18^ Seven items assessed the frequency of anxiety symptoms over the past 2 weeks on a 4-point Likert-scale ranging from 0 (never) to 3 (nearly every day). The total score of GAD-7 ranged from 0 to 21, with increasing scores indicating more severe functional impairments as a result of anxiety.^19^ For the purpose of this study, a GAD-total score of 9 points or greater indicated the presence of anxiety symptoms. The Center for Epidemiology Scale for Depression (CES-D) in Pakistani version was used to identify whether participants had depressive symptoms. The sensitivity and specificity of CES-D has been reported to be very good with a Cronbach’s alpha of 0.88 for general population samples.^20^ Twenty items assessed the frequency of depressive symptoms over the past 2 weeks on a 4-point Likert-scale ranging from 0 (rarely or none of the time) to 3 (most or all of the time). The score range of the CES-D was 0–60 points, and higher scores indicated more severe depressive symptoms.^21^ In our study, CES-D scores greater than 28 points indicated depressive symptoms. The overall Cronbach’s alpha of the entire instrument was calculated to be 0.90. Descriptive statistics were used to report demographic characteristics, COVID-19-related information, and attitude concerning the COVID-19 pandemic. The prevalence of GAD and depressive symptoms was stratified by demographic information, COVID-19-related attitude, and COVID-19-related knowledge with χ^2^/Fischer’s test used to compare the differences between groups. Lastly, univariate and multivariate logistic regression models were performed to explore potential influence factors for GAD, and depressive symptoms during COVID-19 outbreak using crude odds ratio (OR), adjusted odds ratio (AOR), and 95% CI. All data were analyzed using Statistical Package for Social Sciences (SPSS) version 25.0. *P* values of less than 0.05 were considered statistically significant (two-sided tests).

## RESULTS

### General characteristics of participants.

A total of 550 online questionnaires were distributed, out of which 500 forms were completely filled and returned, making the response rate as 90.9%. In a total of 500 participants analyzed in the current survey, greater than two-fifths (*N* = 228) 45.6% were females. There were (*N* = 138) 27.6% individuals aged 41–50 years, and (*N* = 124) 24.8% participants aged 51–60 years. The highest proportion (*N* = 405) 81.0% of our participants were married whereas nearly one-fifths (*N* = 103) 20.6% had no formal education. More than half (*N* = 263) 52.6% earned < 20,000 rupees followed by nearly one-thirds (*N* = 146) 29.2% earning < 50,000 rupees as shown in Table 1. Highest number of responses came from province Punjab (Supplemental Figure).

**Table 1:** General characteristics of the study respondents (*N* = 500)

General characteristics	Frequency, *N* (%)
Age (years)	
< 18 18–30 31–40 41–50 51–60	13 (2.6) 72 (14.4) 153 (30.6) 138 (27.6) 124 (24.8)
Gender	
Male Female	272 (54.4) 228 (45.6)
Educational status	
No formal education Primary school Secondary/higher secondary Undergraduate or more	103 (20.6) 148 (29.6) 142 (28.4) 107 (21.4)
Occupation	
Healthcare worker* Enterprise/institution worker† Teachers/students‡ Unemployed Others§	52 (10.4) 86 (17.2) 25 (5.0) 219 (43.8) 118 (23.6)
Marital status	
Married Single Widowed/Divorced	405 (81.0) 91 (18.2) 4 (0.8)
Monthly income (Rupees)	
< 20,000 21,000–50,000 51,000–100,000 101,000–200,000 > 200,000	263 (52.6) 146 (29.2) 60 (12.0) 25 (5.0) 6 (1.2)
Obtaining constant critical COVID-19 updates	
Yes No	314 (62.8) 186 (37.2)
Time spent focusing on the COVID-19 information every day (hours)	
< 1 1–2 ≥ 3	315 (63.0) 91 (18.1) 94 (18.8)
Fear of contracting COVID-19	
Yes No	326 (65.2) 174 (34.8)
Prevalence of GAD symptoms	
Yes No	127 (25.4) 373 (74.6)
Prevalence of depressive symptoms	
Yes No	94 (18.8) 406 (81.2)
Knowledge levels regarding COVID-19 outbreak	
Do not understand Generally understand Quite understand	88 (17.6) 35 (7.0) 377 (75.4)

GAD = generalized anxiety disorder.

* Included doctors, nurses, and health administrators.

† Included enterprise employees, national/provincial/municipal institution workers, and other relevant staff.

‡ Included teachers or students from universities, middle schools, or elementary schools.

§ Included freelancers, retiree, social workers, and other relevant staff.

### Knowledge, attitude concerning the COVID-19 pandemic, prevalence of GAD, and depressive symptoms among participants.

The mean and SD for the total knowledge score was 5.09 (SD ± 1.85) out of a possible score of 7. The median was 6.00 (inter quartile range [IQR] 5.00–6.00). Over three-quarters (*N* = 377) 75.4% of the respondents quite understood the knowledge regarding COVID-19, followed by (*N* = 35) 7.0% participants who generally understood the knowledge of COVID-19, and nearly one-fifths (*N* = 88) 17.6% who did not understand the knowledge of COVID-19. The number of correct responses of the participants to individual knowledge questions are demonstrated in Table 2. With regards to attitude concerning the COVID-19 pandemic, similar proportions of our respondents spent 1–2 hours (*N* = 91) 18.2% and > 3 hours (*N* = 94) 18.8% focusing on COVID-19, while nearly two-thirds (*N* = 314) 62.8% used social media to obtain constant critical COVID-19 updates. Finally, one-thirds (*N* = 174) 34.8% of our respondents had no fear of contracting SARS-CoV-2 infection. The overall prevalence of GAD and depressive symptoms among our participants was documented to be (*N* = 127) 25.4% and (*N* = 94) 18.8%, respectively.

**Table 2:** Responses of the participants to knowledge items

Knowledge items	Correct responses N (%)	Incorrect responses N (%)	Not sure *N* (%)
Inhalation of droplets from sneezing, coughing, or talking to an infected person could cause infection	442 (88.4)*	12 (2.4)	46 (9.2)
Contact with something contaminated by an infected person could lead to infection	412 (82.4)*	24 (4.8)	64 (12.8)
The incubation period of the virus does not exceed 14 days	345 (69.0)*	29 (5.8)	126 (25.2)
Contact with an asymptomatic person might also lead to infection	238 (47.6)*	150 (30.0)	112 (22.4)
There are already targeted drugs that could cure the disease	275 (55.0)†	96 (19.2)	129 (25.8)
Not all people with COVID-19 will develop to severe cases. Only those who are elderly, have chronic illnesses, and obese are more likely to be severe cases	373 (74.6)*	57 (11.4)	70 (14.0)
The main clinical symptoms of COVID-19 are fever, fatigue, dry cough, and muscle ache	461 (92.2)*	5 (1.0)	34 (6.8)

* Correct response is yes.

† Correct response is no.

### Prevalence of GAD and depressive symptoms stratified by general characteristics, knowledge and, attitude concerning COVID-19 pandemic.

There was a highly statistically significant difference in the prevalence of GAD symptoms by gender (*P* = 0.001) as more than half (*N* = 74) 58.3% of anxious participants were females as shown in Table 3. Generalized anxiety disorder was also significantly associated with marital status (*P* = 0.034), for instance, the majority (*N* = 106) 83.5% of the anxious respondents were married. When comparing GAD symptoms with attitude related to the COVID-19 pandemic, the prevalence of GAD was reported to be significantly higher in patients who obtain constant COVID-19 critical updates (*N* = 89) 70.1% (*P* = 0.049). However, on the contrary, the prevalence of GAD was not significantly correlated with the time spent on focusing COVID-19 (*P* = 0.244). Lastly, nearly three-quarters (*N* = 93) 73.2% of the anxious respondents feared of contracting SARS-CoV-2 infection compared with those who did not (*N* = 34) 26.8% and this difference was observed to be statistically significant (*P* = 0.028). Only (*N* = 28) 22.0% of anxious respondents did not understand the knowledge regarding COVID-19, however, this was not statistically significant.

**Table 3:** Association of GAD and depressive symptoms with general characteristics among study population (*N* = 500)

Study variables	GAD			*P* value		Depressive symptoms		*P* value
	Present	Absent				Present	Absent	
Gender								
Females	74 (58.3)	154 (41.3)		0.001*		52 (55.3)	176 (42.3)	0.036*
Males	53 (41.7)	219 (58.7)				42 (44.7)	230 (56.7)	
Age (years)								
< 18	0 (0.0)	13 (3.5)		0.055†		0 (0.0)	13 (3.2)	0.017†
18–30	17 (13.4)	55 (14.7)				6 (6.4)	66 (16.3)	
31–40	48 (37.8)	105 (28.2)				28 (29.8)	125 (30.8)	
41–50	36 (28.3)	102 (27.3)				30 (31.9)	108 (26.6)	
51–60	26 (20.5)	98 (26.3)				30 (31.9)	94 (23.2)	
Marital status								
Married	106 (83.5)	299 (80.2)		0.034†		83 (88.3)	322 (79.3)	0.001†
Single	18 (14.2)	73 (19.6)				8 (8.5)	83 (20.4)	
Widowed/Divorced	3 (2.4)	1 (0.3)				3 (3.2)	1 (0.2)	
Education status								
No formal education	28 (22.0)	75 (20.1)		0.714*		15 (16.0)	88 (21.7)	0.150*
Primary school	37 (29.1)	111 (29.8)				34 (36.2)	114 (28.1)	
Secondary/higher secondary	39 (30.7)	103 (27.6)				21 (22.3)	121 (29.8)	
Undergraduate or more	23 (18.1)	84 (22.5)				24 (25.5)	83 (20.4)	
Monthly income (PKR)								
< 20,000	61 (48.0)	202 (54.2)		0.214†		53 (56.4)	210 (51.7)	0.371†
< 50,000	45 (35.4)	101 (27.1)				29 (30.9)	117 (28.8)	
< 100,000	17 (13.4)	43 (11.5)				7 (7.4)	53 (13.1)	
100,000–200,000	3 (2.4)	22 (5.9)				3 (3.2)	22 (5.4)	
> 200,000	1 (0.8)	5 (1.3)				2 (2.1)	4 (1.0)	
Occupation								
Unemployed	63 (49.6)	156 (41.8)		0.524*		47 (50.0)	172 (42.4)	0.225†
Healthcare worker	14 (11.0)	38 (10.2)				6 (6.4)	46 (11.3)	
Enterprise/institution workers	20 (15.7)	66 (17.7)				11 (11.7)	75 (18.5)	
Teachers/students	6 (4.7)	19 (5.1)				4 (4.3)	21 (5.2)	
Others	24 (18.9)	94 (25.2)				26 (27.7)	92 (22.7)	
Obtaining constant critical COVID-19 updates								
Yes	89 (70.1)	225 (60.3)		0.049*		66 (70.2)	248 (61.1)	0.099*
No	38 (29.9)	148 (39.7)				28 (29.8)	158 (38.9)	
Time spent focusing on the COVID-19 (hours)								
< 1	77 (60.6)	238 (63.8)		0.244*		60 (63.8)	255 (62.8)	0.947*
1–2	20 (15.7)	71 (19.0)				16 (17.0)	75 (18.5)	
≥ 3	30 (23.6)	64 (17.2)				18 (19.1)	76 (18.7)	
Fear of contracting COVID-19								
Yes	93 (73.2)		233 (62.5)		0.028*	64 (68.1)	262 (64.5)	0.515*
No	34 (26.8)		140 (37.5)			30 (31.9)	144 (35.5)	
Knowledge levels regarding COVID-19 outbreak								
Do not understand	28 (22.0)		60 (16.1)		0.063*	9 (9.6)	79 (19.5)	0.072*
Generally understand	4 (3.1)		31 (8.3)			8 (8.5)	27 (6.7)	
Quite understand	95 (74.8)		282 (75.6)			77 (81.9)	300 (73.9)	

GAD = generalized anxiety disorder; PKR = Pakistani rupee.

* Chi-square test.

† Fisher’s exact test.

When stratifying depressive symptoms by demographic factors, a statistically significant difference in depression was observed by gender (*P* = 0.036) as greater than two-fifths (*N* = 42) 44.7% of depressive participants were males. Equal proportions (*N* = 30) 31.9% of depressive respondents were aged 41–50 years and 51–60 years, whereas none of the participants aged < 18 years was positive for depressive symptoms (*P* = 0.017). A huge difference in the prevalence of depression was documented when stratifying by marital status as most of the respondents (*N* = 83) 88.3% positive for depressive symptoms were married (*P* = 0.001). Lastly, no significant association was reported between prevalence of depression and attitude as well as knowledge regarding COVID-19.

### Association of influence factors with GAD and depressive symptoms during COVID-19 outbreak.

In the univariate logistic regression models (Table 4), gender (OR = 1.986, 95% CI: 1.320–2.987), obtainment of constant critical updates regarding COVID-19 (OR = 1.541, 95% CI: 0.999–2.375), and fear of contracting COVID-19 (OR = 1.644, 95% CI: 1.053–2.565) were significantly correlated with GAD. Moreover, gender (OR = 0.618, 95% CI: 0.393–0.971) and age (OR = 3.511, 95% CI: 1.383–8.910) were significantly associated with depressive symptoms. Participants who did not understand the knowledge regarding COVID-19 had significantly higher odds of developing depression (OR = 2.253, 95% CI: 1.082–4.692). In the multivariate logistic regression models after adjusting for background characteristics, the association of GAD with gender (AOR = 2.172, 95% CI: 1.306–3.613), and obtainment of constant critical updates (AOR = 1.770, 95% CI: 1.037–3.019) further strengthened. Participants with a monthly income of < 50,000 rupees (AOR = 4.263, 95% CI: 1.034–17.586), and < 100,000 rupees (AOR = 4.450, 95% CI: 1.058–18.726) were more likely to develop GAD than those who earned > 200,000 rupees. Similarly, participants under 30 years were associated with a higher risk of depressive symptoms (AOR = 2.995, 95% CI: 1.057–8.489) than those aged > 30 years. Lastly, participants with no formal education (AOR = 3.053, 95% CI: 1.197–7.784) were at the highest risk of developing depressive symptoms.

**Table 4:** Univariate and multivariate logistic regression model for GAD and depression (*N* = 500)

Variables	GAD		Depressive symptoms	
	OR (95% CI)	AOR (95% CI)	OR (95% CI)	AOR (95% CI)
Gender				
Male	1.000	1.000	1.000	1.000
Female	1.986 (1.320–2.987)*	2.172 (1.306–3.613)*	0.618 (0.393–0.971)*	0.627 (0.354–1.111)
Age (years)				
< 18	–	–	–	–
18–30	1.165 (0.582–2.334)	0.997 (0.438–2.268)	3.511 (1.383–8.910)*	2.995 (1.057–8.489)*
31–40	1.723 (0.993–2.989)	1.691 (0.869–3.290)	1.425 (0.797–2.564)	1.215 (0.600–2.463)
41–50	1.330 (0.748–2.366)	1.156 (0.619–2.157)	1.149 (0.645–2.045)	1.074 (0.569–2.028)
51–60	1.000	1.000	1.000	1.000
Education status				
No formal education	1.363 (0.724–2.569)	1.683 (0.700–4.045)	1.696 (0.833–3.455)	3.053 (1.197–7.784)*
Primary school	1.217 (0.673–2.202)	1.399 (0.633–3.095)	0.970 (0.535–1.756)	1.695 (0.762–3.769)
Secondary/higher secondary	1.383 (0.766–2.496)	1.340 (0.661–2.717)	1.666 (0.871–3.188)	2.916 (1.331–6.385)
Undergraduate or more	1.000	1.000	1.000	1.000
Monthly income (PKR)				
< 20,000	2.215 (0.641–7.651)	1.775 (0.394–7.898)	0.540 (0.156–1.873)	0.535 (0.114–2.516)
< 50,000	3.267 (0.930–11.477)	4.263 (1.034–17.586)*	0.550 (0.154–1.965)	0.406 (0.094–1.754)
< 100,000	2.899 (0.766–10.968)	4.450 (1.058–18.726)*	1.032 (0.244–4.362)	0.890 (0.193–4.109)
100,000–200,000	1.467 (0.125–17.213)	1.440 (0.116–17.845)	0.273 (0.034–2.188)	0.306 (0.032–2.917)
> 200,000	1.000	1.000	1.000	1.000
Occupation				
Unemployed	1.582 (0.926–2.701)	1.570 (0.755–3.268)	1.034 (0.602–1.778)	0.762 (0.356–1.629)
Healthcare worker	1.443 (0.675–3.083)	1.402 (0.526–3.379)	2.167 (0.833–5.634)	2.262 (0.688–7.431)
Enterprise/institution workers	1.187 (0.606–2.323)	0.926 (0.441–2.086)	1.927 (0.894–4.154)	1.558 (0.613–3.961)
Teachers/students	1.237 (0.445–3.435)	0.675 (0.209–2.181)	1.484 (0.468–4.707)	1.221 (0.329–4.529)
Others	1.000	1.000	1.000	1.000
Obtaining constant critical COVID-19 updates				
Yes	1.541 (0.999–2.375)*	1.770 (1.037–3.019)*	0.666 (0.410–1.082)	0.587 (0.330–1.045)
No	1.000	1.000	1.000	1.000
Time spent focusing on COVID-19 (hours)				
< 1	1.000	1.000	1.000	1.000
1–2	1.149 (0.657–2.008)	0.743 (0.403–1.371)	1.103 (0.600–2.027)	1.239 (0.639–2.401)
≥ 3	1.664 (0.861–3.216)	1.009 (0.567–1.797)	0.993 (0.553–1.785)	1.467 (0.756–2.847)
Fear of contracting COVID-19				
Yes	1.644 (1.053–2.565)*	1.593 (0.965–2.631)	0.853 (0.528–1.377)	0.725 (0.420–1.252)
No	1.000	1.000	1.000	1.000
Knowledge levels regarding COVID-19 outbreak				
Do not understand	1.385 (0.836–2.296)	1.600 (0.760–3.367)	2.253 (1.082–4.692)*	2.457 (0.968–6.234)
Generally understand	0.383 (0.132–1.113)	0.365 (0.118–1.133)	0.866 (0.379–1.982)	0.884 (0.344–2.272)
Quite understand	1.000	1.000	1.000	1.000

AOR = adjusted odds ratio; GAD = generalized anxiety disorder; OR = crude odds ratio; PKR = Pakistani rupee.

* Indicates significant *P* values of < 0.05.

## DISCUSSION

Our study showed a high prevalence of depression and GAD symptoms among Pakistani adults during the second wave of the COVID-19 outbreak. Our findings provide data for accurately understanding the mental health implications of COVID-19 pandemic on citizens to help physicians initiate appropriate interventions. We found that 25.4% of the participants suffered from GAD whereas 18.8% showed symptoms of depression, indicating the psychiatric implications of this pandemic. Another study conducted by Khan et al., demonstrated 41.2% of the participants to suffer from poor psychological well-being.^6^ According to the findings of a pooled analysis led by Bareeqa et al., stress was the most prevalent (48.1%) psychological consequence of the COVID-19 pandemic, followed by depression (26.9%) and anxiety (21.8%).^22^ A very recent study conducted by Mamun et al., in Bangladesh reported 33.3% of the participants to suffer from depression.^23^ A possible reason for these mental health problems could be the increased exposure to COVID-19-related misinformation on various news and media channels, gas-lighting their concerns. Among all, social media also played a vital role in propagating fear and panic among people during these uncertain times. Information with little or no credibility spread rapidly through these platforms, and contributed significantly to the rising fear.^24^ This is also supported by our findings that showed that citizens who obtained constant critical COVID-19 updates were 1.8 times more likely to experience GAD on multivariate logistic regression models. A previous study reported a high proportion (90%) of the participants to believe that unauthentic information through social media is adding to the panic about COVID-19.^6^ Labeling COVID-19 as an “exclusive threat” by the media has also had significant mental health repercussions on the population.^25^ Checking of critical COVID-19 updates repeatedly thus seemed to be an important contributor to stress among people, leading to increased fear of contracting the virus.

A significant proportion of female participants experienced symptoms related to anxiety, similar to the findings of a Chinese study, in which women were shown to be more vulnerable to psychological disturbances than men.^26^ A possible cause for this finding in women is domestic violence and physical abuse, whereas it is usually financial and economic losses in case of men. Comorbidities and the imposed restrictions have contributed significantly in aggravating these factors.^27^ Other studies conducted in China, Iraq, and Spain also reported female gender to be an important predictor of depression, anxiety, and stress compared with males during the COVID-19 pandemic.^28^^–^^30^ Another possible explanation of these findings is that women are the primary caregivers in the households who are overburdened with routine household work. In addition, they also take care of the responsibilities related to children, and male members of the family who are stuck at home because of the lockdown. This makes females more vulnerable to poor psychological well-being.^6^

An interesting finding was the high prevalence of depressive symptoms in males as depression in males usually goes undiagnosed as a result of the various coping mechanisms they use.^31^ A significant number of participants < 30 years of age also reported experiencing depressive symptoms. The early onset depression has a strong association with low levels of self-esteem. People in this age group frequently experience low social support and resilience while insensitive parenting and unavailability can also contribute equally toward early onset depression.^32^^,^^33^

After univariate logistic regression analysis, we found that participants who did not understand information about COVID-19 had higher odds of developing depression. This included several factors such as sanitation, transmission of the virus, treatment, and protective and preventive measures like social distancing and hand hygiene. This inadequacy in knowledge was fueled by misinformation and led to incidents of panic buying, hoarding of supplies, stigmatization, xenophobia, and discrimination, resulting in poor mental health.^34^ We found that lesser proportion of population correctly answered about the transfer of virus from an asymptomatic person, and only 54.9% knew that there are no targeted drugs available. This could have led to the increased incidence of self-medication during the pandemic, which resulted in panic buying of drugs and a spike in drug prices leading to lesser people who can afford it, thus further affecting their mental health.^35^ Factors such as low psychosocial support and low perceived likelihood of survival also play an important role in determining an individual’s mental state.^36^ According to the findings of a study, a major proportion (68%) of the sample population were worried about contracting the disease even with preventive measures, 6% were worried about surviving after the infection and no effects of treatment.^6^ Increased number of information campaigns organized by government agencies in local, easy to understand language could decrease the dread and anxiety caused to people, thus having a positive impact on their mental health. Provision of credible information by doctors and media will also go a long way.^37^

Participants with a monthly income of > 50,000 rupees and > 100,000 rupees were more likely to develop GAD than those who earned > 200,000 rupees. This could be related to stress caused by decreased wages with many family members to financially support, and other factors that marked the early months of the COVID-19 pandemic. Pakistan is a lower middle-income country with limited resources, and the majority of the population struggles to make their ends meet. A recent study indicated 41% of the participants to suffer on financial sources other than salary including savings (15.4%), business earning (12.6%), support from friends and family (7.0%), no source (3.6%), loan (1.7%), and pension (0.9%).^6^ Furthermore, the imposed restrictions resulted in factory shutdowns and pay cuts for the workers with some being laid off their jobs. This led to rising disparities among people, rendering them desperate for financial aid, fueling the tension.^38^

Participants under 30 years were associated with a higher risk of depressive symptoms, than those > 30 years, a finding similar to a study in Taiwan during the SARS outbreak.^39^ Lastly, participants with no formal schooling were at the highest risk of developing depressive symptoms. Demonstration and pictorial representation of COVID-19 norms, hygiene, and safety practices can also help tremendously in conveying information to the uneducated population and curb the resulting panic. These uneducated participants are also likely to belong to the poor socioeconomic class. Interventions like introducing free of cost mental health screening as part of routine health checkup and imparting knowledge through paraprofessional workers in the primary care setting of public hospitals will result in more people availing the services and better facilitation of care.^40^ Telemedicine therapy is also a recently discovered avenue that is rapidly expanding and can contribute significantly in delivering information to geographically remote locations while strengthening primary care delivery and reducing social disparities.^41^

Several appropriate interventions are recommended: first, people need to check whether the provided information is from a credible source and try to limit their social media exposure. Maintaining a healthy relationship with family and friends and sharing feelings can help alleviate stress tremendously. Psychological interventions should also be implemented for the vulnerable population, including family of the affected and individuals with chronic conditions. Keeping oneself busy and maintaining a normal working rhythm and exercising can also help keep stressful thoughts away. The government also needs to step in and help people find alternatives to sustain themselves financially during these difficult times, by protecting the vulnerable and using the limited supplies judiciously.^42^ Some of the coping strategies recommended to reduce psychological distress includes exercise during lockdown, spending time with family and friends, eating healthy food, adequate sleep, participating in social welfare, and spending time on hobbies. These strategies have been reported to be effective in few studies conducted in the Poland and China.^43^^,^^44^

## LIMITATIONS

The limitations of the study included a cross-sectional design so the data and analyses were difficult to make causal inferences. Secondly, the study was conducted by online system because of COVID-19 outbreak, hence sampling of our study was voluntary. Therefore, the possibility of selection bias should be considered. Thirdly, this study measured psychiatric symptoms mostly using self-reported questionnaires, and did not establish a clinical diagnosis. Structured clinical interviews and functional neuroimaging are the gold standard for establishing a psychiatric diagnosis.^45^ Despite these limitations, this study was able to report anxiety and depression among the general population during the COVID-19 pandemic, however, the previous psychological conditions of the study participants before the pandemic could not be assessed and cannot be reported.

## CONCLUSION

In conclusion, we identified a potential threat to mental health among Pakistani adults during the second wave of the COVID-19 pandemic. Our study indicates a relatively high prevalence of anxiety and depressive symptoms among people during the COVID-19 outbreak. We identified several demographic and other variables causing a negative impact on the mental health, with the social isolation and other negative effects of the pandemic acting as aggravating factors. The associated mental health hazards of this pandemic need radical interventions to combat the psychological issues, especially targeting the vulnerable population. We need a better understanding of the multiple health implications of COVID-19 to devise measures to overcome it.

## Supplemental Material


Supplemental materials
