# Supplementary figures and images for: Generalized Anxiety Disorder and Depressive Symptoms among Pakistani Population during the Second Wave of the COVID-19 Pandemic: A Regression Analysis
Source: Am J Trop Med Hyg. 2021 Aug 30;105(4):915–23. doi: 10.4269/ajtmh.21-0380 (PMC8592168; doi:10.4269/ajtmh.21-0380)

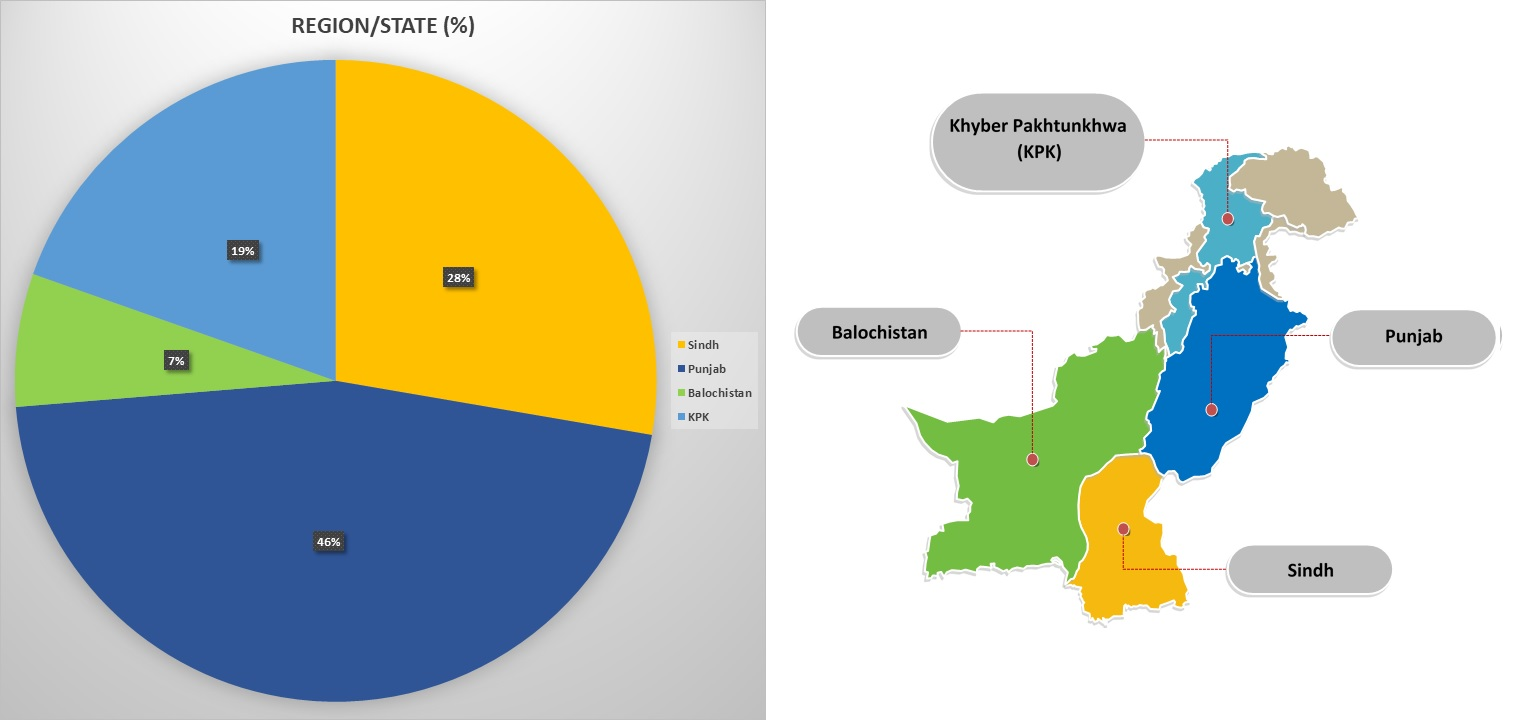

Supplement: Supplementary file 1 [file tpmd210380.SD1.tif]
